# Intracranial Solitary Fibrous Tumor: A Mimicker of Meningioma

**DOI:** 10.5334/jbr-btr.858

**Published:** 2015-09-15

**Authors:** W. Van Dessel, G. Wilms

**Affiliations:** 1 Department of Radiology, Universitaire Ziekenhuizen Leuven, Leuven, Belgium

In September 2009, a 56-year old woman presented to our hospital with meningitis-like complaints. A CT scan demonstrated a small intracranial lesion in the posterior fossa. MRI, performed for further evaluation for this lesion, showed a broad dural-based implantation on the lower surface of the tentorium cerebelli, and a marked enhancement after administration of intravenous contrast (arrow in Fig. A). The diagnosis of a small tentorial meningioma was made. A control CT scan six months later showed no volume increase, after which she was lost to follow up. In October 2011, a new CT scan performed in the context of a minor head trauma, showed a significant volume-increase of this lesion. A new MRI scan was performed which confirmed this. The scan also demonstrated the development of a slight mass-effect on the left cerebellar hemisphere together with minor tumor invasion of the cerebellum and minimal surrounding edema. The tumor showed a heterogeneous aspect on T2 weighted imaging with some small cystic foci (Fig. B). After administration of Gadolinium, a heterogeneous enhancement was present (Fig. C). The preferred diagnosis remained that of a meningioma. Since the tumor appeared to be progressive, surgery was performed with complete resection. Pathological examination of the specimen revealed the lesion to be a meningeal solitary fibrous tumor with malignant features.

**Figures A–C:**
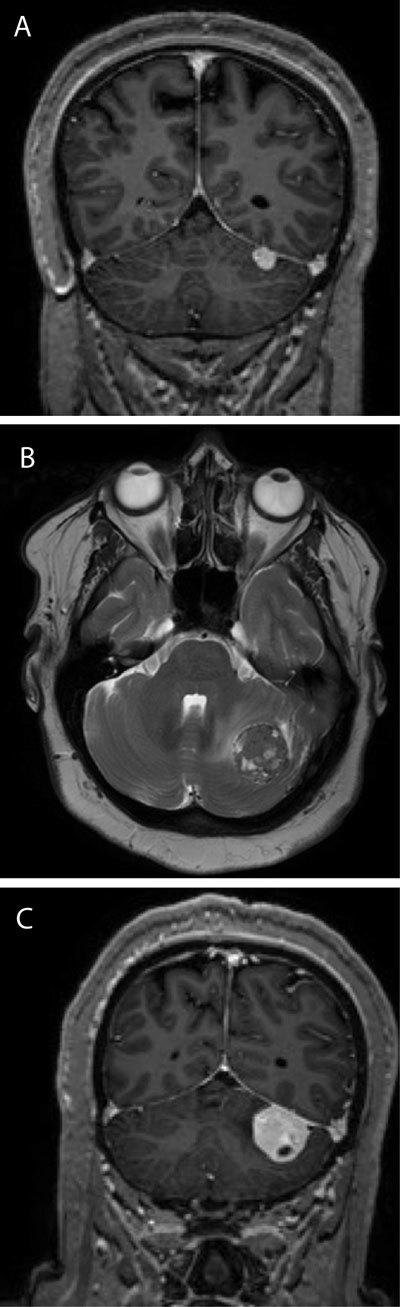


## Comment

A solitary fibrous tumor (SFT) is a spindle-cell neoplasm of mesenchymal origin, that occurs in a variety of locations throughout the body most frequently the pleura. An intracranial location is rather rare. When present, they are often found along the dura or within the ventricles. They manifest around the 5^th^ or 6^th^ decade, with predominance for women. They have benign and malignant variants. The main differential is a meningioma.

SFT’s are iso- to hyperdense well-circumscribed lesions on CT imaging, sometimes with small calcifications. Smooth erosions of the adjacent skull can be seen, this in contrast to the hyperostosis which is often found in meningiomas. On MR imaging, SFT’s demonstrate heterogeneous signal intensity on T2 weighted imaging, with an heterogeneous contrast enhancement. Areas of low signal on T2 imaging that enhance avidly after administration of contrast are suggestive for SFT, this in contrast to meningiomas, which tend to show a more homogeneous enhancement after contrast administration. A dural tail can be present, though not always and is certainly not pathognomonic. Regions of restricted diffusion can be seen, representing hypercellular areas. The presence of flow voids is another feature that is often encountered. On MR spectroscopy raised lipid and lactate peaks are observed, which can be helpful in the differentiation with meningiomas that typically show elevated alanine and glutamate-glutamic acid peaks.

As mentioned above, the main differential diagnosis is a meningioma, which should be on the number one position of everybody’s differential list when encountering a dural-based lesion. Next to the solitary fibrous tumor, other less common dural-based lesions are hemangiopericytoma, leiomyoma (or –sarcoma), lymphoma, sarcoid, and other very rare conditions.

## Competing Interests

The authors declare that they have no competing interests.

## References

[1] Smith AB, Horkanyne-Szakaly I, Schroeder JW, Rushing EJ (2014). Mass lesions of the dura: beyond meningioma – radiologic-pathologic correlation. RadioGraphics.

